# Transient Receptor Potential Vanilloid 1 is essential for cisplatin-induced heat hyperalgesia in mice

**DOI:** 10.1186/1744-8069-6-15

**Published:** 2010-03-05

**Authors:** Lauren E Ta, Allan J Bieber, Susan M Carlton, Charles L Loprinzi, Philip A Low, Anthony J Windebank

**Affiliations:** 1 Program in Molecular Neuroscience, Mayo Graduate School, Mayo Clinic, College of Medicine, Rochester, MN 55905, USA; 2 Department of Neuroscience, Mayo Clinic, College of Medicine, Rochester, MN 55905, USA; 3 Department of Neurology, Mayo Clinic, College of Medicine, Rochester, MN 55905, USA; 4 Division of Medical Oncology, Mayo Clinic, College of Medicine, Rochester, MN 55905, USA; 5 Department of Neuroscience and Cell Biology, University of Texas Medical Branch, Galveston, TX 77555, USA

## Abstract

**Background:**

Cisplatin is primarily used for treatment of ovarian and testicular cancer. Oxaliplatin is the only effective treatment for metastatic colorectal cancer. Both are known to cause dose related, cumulative toxic effects on the peripheral nervous system and thirty to forty percent of cancer patients receiving these agents experience painful peripheral neuropathy. The mechanisms underlying painful platinum-induced neuropathy remain poorly understood. Previous studies have demonstrated important roles for TRPV1, TRPM8, and TRPA1 in inflammation and nerve injury induced pain.

**Results:**

In this study, using real-time, reverse transcriptase, polymerase chain reaction (RT-PCR), we analyzed the expression of TRPV1, TRPM8, and TRPA1 induced by cisplatin or oxaliplatin in vitro and in vivo. For in vitro studies, cultured E15 rat dorsal root ganglion (DRG) neurons were treated for up to 48 hours with cisplatin or oxaliplatin. For in vivo studies, trigeminal ganglia (TG) were isolated from mice treated with platinum drugs for three weeks. We show that cisplatin and oxaliplatin-treated DRG neurons had significantly increased in TRPV1, TRPA1, and TRPM8 mRNA expression. TG neurons from cisplatin treated mice had significant increases in TRPV1 and TRPA1 mRNA expression while oxaliplatin strongly induced only TRPA1. Furthermore, compared to the cisplatin-treated wild-type mice, cisplatin-treated TRPV1-null mice developed mechanical allodynia but did not exhibit enhancement of noxious heat- evoked pain responses. Immunohistochemistry studies showed that cisplatin-treated mice had no change in the proportion of the TRPV1 immunopositive TG neurons.

**Conclusion:**

These results indicate that TRPV1 and TRPA1 could contribute to the development of thermal hyperalgesia and mechanical allodynia following cisplatin-induced painful neuropathy but that TRPV1 has a crucial role in cisplatin-induced thermal hyperalgesia in vivo.

## Background

Painful peripheral neuropathy is the principle dose-limiting factor requiring discontinuation of chemotherapy with platinum-based drugs such as cisplatin and oxaliplatin [1]. Cisplatin is widely used for treatment of solid tumors especially against testicular, ovarian, and bladder cancers [2,3]. Oxaliplatin is a third generation platinum analogue which is highly effective for metastatic colorectal cancer [4-6]. Platinum-based drugs presumably exert their antitumor activity by binding to DNA and distorting the helical structure in a way that inhibits transcription [7] and induces apoptotic cell death through DNA damage recognition pathways [8,9]. Up to thirty to forty percent of cancer patients that receive platinum agents develop pain and sensory changes, resulting in chronic dose-limiting peripheral neuropathy [10]. In addition, oxaliplatin induces distinctive acute cold-associated dysesthesia in up to 80% of patients [11]. Current pain treatments for chemotherapeutic neuropathy have limited effectiveness, a fact that reflects our poor understanding of the underlying pain mechanisms.

Rodent models of platinum-drug induced painful neuropathy have been developed to elucidate the pain mechanisms caused by platinum drugs [12-14]. Pharmacological studies using these models indicate that the mechanisms underlying mechanical and thermal hyperalgesia behaviors following platinum drugs are complex [13,15-17]. Neurophysiological and biochemical studies show that activation and sensitization of nociceptors plays a key role in pathological pain behaviors following platinum drugs [18-20]. The putative non-selective cation channels, transient receptor potential vanilloid 1 (TRPV1), TRPA1, and TRPM8 are the primary detectors involved in chemical and thermal evoked pain sensation [21] but the precise contribution of TRPV1, TRPA1, and TRPM8 in sensitizing nociceptors after cisplatin and oxaliplatin drugs in vivo is not known.

TRPV1 is a capsaicin receptor that is activated by painful chemical stimuli, by noxious heat (activated at 42°C), and inflammation, but not by nerve ligation injury [22-24]. TRPA1 has a functional role in pain and neurogenic inflammation resulting from channel activation to a variety of compounds including pungent agents, irritant chemicals, reactive oxygen and nitrogen species, and products of oxidative stress-induced lipid peroxidation [25-33]. TRPA1 has been shown to co-localize with TRPV1 in subpopulations of DRG and TG neurons [34,26]. Studies with TRPA1 knockout mice have demonstrated loss of mustard oil responses in vitro and in vivo [28,35], and in vivo development of bradykinin-induced mechanical hypersensitivity [35]. TRPA1 has also been implicated as a sensor for painfully cold temperatures (<17°C) [34,36,37,35] but these findings are not consistent with others [26,38,28]. TRPM8 is activated either by menthol or cool temperatures (<27°C) [39,40] and is involved in cold-evoked responses in vivo [41,42]. Although the expression of these channels is increased in other pain models [43-47], it is not yet known whether platinum drug induced TRPV1, TRPA1, and TRPM8 expression and functional changes contribute to altered neuronal sensitivity and excitability in toxic neuropathy.

In the present study, we have used a recently developed mouse model of cisplatin and oxaliplatin-induced painful neuropathy [48] to investigate the molecular mechanisms involved in hyperalgesia after platinum drug treatment. First, we demonstrate that treatment with platinum drugs results in the up-regulation of TRPV1, TRPA1, and TRPM8 mRNA in cultured dorsal root ganglion (DRG) neurons and that a similar up-regulation occurs with TRPV1 and TRPA1 following in vivo treatment with cisplatin although cisplatin-treated mice had no change in the proportion of TRPV1-immunopositive TG neurons. Oxaliplatin treatment results in up-regulation of only TRPA1 in vivo. We also illustrate that up-regulation of TRPV1 and TRPA1 mRNA reflects increases in TRPV1 and TRPA1 responsiveness in the nociceptors that contribute to the molecular mechanisms of the thermal hyperalgesia and mechanical allodynia observed in cisplatin-treated mice. Second, we demonstrate that TRPV1 is required for mediating heat, but not mechanical hyperalgesia in cisplatin-induced nerve injury.

## Results and Discussion

### Cisplatin and oxaliplatin induced up-regulation of TRPV1, TRPM8 and TRPA1 mRNA in vitro

Several cellular effects of platinum-based drugs have been described which may be involved in the development of neurotoxicity, including platinum-DNA binding [49,50] leading to cell cycle alterations and neuronal death [51], neurite outgrowth inhibition [52,53], vesicular axonal transport and alteration of microtubule assembly [54,55]. It remains unclear whether these changes are the primary factors that trigger a cascade of events leading to neuronal damage which contributes to neuropathic pain. In this study we directly measure the transcripts of the nociceptive TRP channels in sensory neurons that are likely to be involved in altered pain response in platinum drug -induced neuropathy.

**Figure 1:**
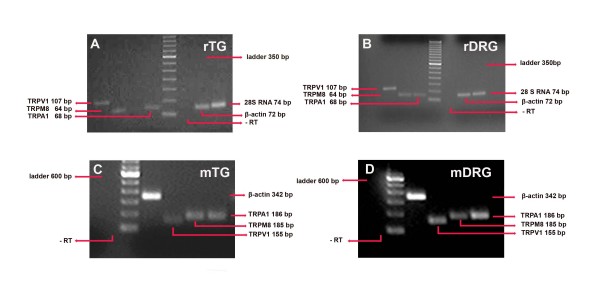
**Expression of TRPV1, TRPM8, and TRPA1 mRNA in Rat and Mouse TG and DRG Tissues**. Reverse transcribed RNA (0.2 μg) from TG and DRG tissues of control Sprague Dawley rats (rTG, rDRG) and C57BL/6J wild-type mice (mTG, mDRG) was added to the reaction mixtures and PCR products amplified in 30 cycles. Control reactions included PCR reactions without reverse transcriptase (-RT). The TRPV1, TRPM8, TRPA1, 28S RNA, and β Actin products were separated on agarose gels and stained with ethidium bromide. Molecular markers (600 and 300 bp ladder) are as shown.

To elucidate the roles of the hot and cold thermoreceptors in sensory neurons, we first assessed TRPV1, TRPM8, and TRPA1 mRNA expression in rat and mouse peripheral tissues. Standard RT-PCR of TRP mRNA from trigeminal ganglion (TG) and DRG tissues from naive rat and wild-type mice yielded PCR products of expected sizes (Figure 1). DRG and TG from rat and mouse tissues were consistently positive for all three TRP receptors. 28S RNA mRNA served as control for the normalization of expression levels.

**Figure 2:**
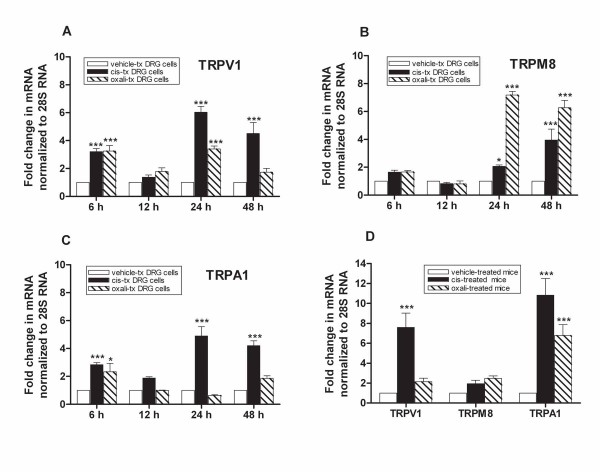
**Platinum drug induced TRPV1 and TRPA1 mRNA up-regulation in vitro and in vivo**. In vitro and in vivo expression of TRV1, TRPM8 and TRPA1 mRNA using real-time RT-PCR are shown. Specific TaqMan primers and probe sets were used for the rat or mouse mRNA of TRPV1, TRPM8, TRPA1, and 28S RNA. The mRNA levels of TRP ion channels are illustrated after normalization to 28S RNA using the 2^-ΔΔCT ^method [87] and presented as the ratio of fold change compared to vehicle-treated cells or tissues. In vitro fold increases of TRV1 **(A) **TRPM8 **(B) **and TRPA1 **(C) **mRNA from rat DRG neurons treated with cisplatin, oxaliplatin, or vehicle across time points 6, 12, 24 and 48 h are shown. Data represents means ± S.E.M from cDNA prepared from triplicate RNA samples. **(D) **In vivo fold increases of TRPV1, TRPM8 and TRPA1 mRNA from TG tissues from cisplatin and oxaliplatin-treated mice compared to vehicle-treated mice after a 3-week drug treatment are shown. Each sample was from pooled TG from three C57BL/6J wild-type mice after 3-week treatment with cisplatin, oxaliplatin or vehicle. Data represents means fold increases ± SEM from cDNA prepared from triplicate RNA samples, (***P < 0.001, **P < 0.01, *P < 0.05, two-way ANOVA, post-hoc analysis).

To determine whether the neurotoxic effects on the peripheral nervous system from platinum drug exposure induces changes in the expression of TRPV1, TRPM8, and TRPA1 mRNA, we used a previously described cell culture model [50]. Rat E15 DRG neurons were grown in equimolar concentrations (6.7 μM) of cisplatin or oxaliplatin, or with vehicle alone. Cells were harvested at 6, 12, 24, and 48 h after treatment and processed for TaqMan RT-PCR of TRPV1, TRPM8, and TRPA1 mRNA. Gene expression levels at each time point were normalized to 28 S RNA and presented as a fold induction compared to the expression level in vehicle-treated cells. This quantitative approach enabled comparisons of the relative changes in expression of each TRP mRNA in response to platinum drugs at varying time points. Cisplatin induced significant up-regulation of TRPV1 and TRPA1 mRNA (3-fold) in cultured DRG neurons at 6 h (Figure 2A and 2C). The expression of both TRPV1 and TRPA1 transcripts continued to show increases (6 and 4.5-fold, respectively) at 24 and 48 h (Figure 2A and 2C). Cisplatin also induced up-regulation of TRPM8 mRNA (2 and 4 fold, respectively) at 24 h and 48 h (Figure 2B). The transient changes of mRNA levels at 6 h could reflect a reversible short-term neuronal response to the initial drug effects. These TRP receptors could arguably be the nocisensors that signal neuronal damage for protective responses. With prolonged drug exposure, sustained increase of the TRP transcripts expression may result from increased long-term neuronal excitability. These data demonstrate a time dependent transcriptional modulation of the expression pattern of TRP channels on DRG neurons following exposure to cisplatin. We found a similar effect of platinum drugs on the levels of neurotoxicity in DRG neurons in a previous in vitro study [50]. We previously demonstrated that both cisplatin and oxaliplatin caused very high levels of platinum-DNA adduct formation in DRG neurons in a dose and time-dependent manner but the levels produced by oxaliplatin were 2-3 times less than those produced by equimolar doses of cisplatin [50]. In the present study, oxaliplatin mirrors cisplatin with an early increase of TRPV1 and TRPA1 but interestingly, higher increases of TRPM8 were detected at 24 and 48 h (7.2 and 6.2-fold, respectively). These observations reveal distinct underlying mechanisms of TRP channel transcriptional regulation by cisplatin and oxaliplatin in DRG neurons.

### Cisplatin induced up-regulations of TRPV1 and TRPA1 mRNA in vivo

To examine whether this phenotype is recapitulated in vivo, we used TaqMan RT-PCR to look at changes in TRP channel gene expression in mouse TG tissues harvested after 3 weeks treatment with platinum drugs [48]. The use of TG allows the isolation of larger amounts of tissue than DRG and TG was therefore used as a surrogate tissue to minimize the number of animals sacrificed. In this pain model [48], mice were treated with daily intraperitoneal injection of platinum drugs for 5 days, followed by 5 days of rest, for two cycles. Total cumulative doses of 23 mg/kg cisplatin and 30 mg/kg oxaliplatin were used; these doses approximate human therapeutic doses relative to body weight. Following two treatment cycles, cisplatin-treated mice developed heat hyperalgesia and mechanical allodynia. In contrast, oxaliplatin-treated mice exhibited only cold hyperalgesia and mechanical allodynia [48]. In vivo expression data shown in Figure 2D demonstrates that cisplatin treated-mice developed significant increases of TRPV1 and TRPA1 mRNA (7.6 and 11-fold, respectively). In contrast, oxaliplatin-treated mice exhibited a significant increase only in TRPA1 mRNA (6.7-fold). While our results illustrate that changes in the relative levels of TRPV1 and TRPA1 mRNA in vivo were similar to those observed in vitro (Figure 2), the increase in TRPM8 mRNA that was observed in oxaliplatin-treated mice was less than that seen in vitro. This discrepancy might simply be due to tissue and species-specific differences. Moreover, given that we are comparing the tissue response of ganglia isolated in vivo to culture neurons isolated from embryonic ganglia, the observed differences could also be affected by the distinct developmental age of the preparations or the neuronal environment.

Our present study shows that mice with oxaliplatin-induced cold hyperalgesia had little change in TRPM8 transcripts expression. Furthermore, a previous behavioral study reported that TRM8^-/- ^mice still respond vigorously to a 0°C cold plate [41] suggesting that another cold-sensitive channel may be involved in this oxaliplatin-induced cold pain model. In contrast to our finding, a recent report showed that mice with oxaliplatin-induced cold allodynia had increased TRPM8 mRNA expression [56]. This discrepancy might be due to the use of different oxaliplatin concentrations (3 mg/kg vs 30 mg/kg), mouse age differences (6 weeks vs 14 weeks), or differences in the cold testing (acetone vs cold plate) or mRNA measurement methods (standard vs quantitative RT-PCR). While there is strong evidence supporting the role of TRPM8 in cold sensation and cooling substances. The presence and alteration of other ion channels activated at noxious cold suggests the presence of TRP-independent mechanisms in oxaliplatin-induced cold hypersensitivity [57-60]. Future studies can address the extent of modulation of other ion channels by direct action of platinum drugs.

Previous behavioral studies have shown that TRPA-/- mice have normal cold sensitivity [28]. However, we demonstrate that cisplatin-treated mice have a robust increase of TRPA1 mRNA without evidence of cold hyperalgesia suggesting that TRPA1 is unlikely to play a role in cold nociception in this model. Others have shown that rats administered with antisense TRPA1 intrathecally showed inhibition to inflammation and spared nerve injury-induced cold hyperalgesia [36,37]. It remains to be determined whether these differences are related to the differences in species or the nerve injury model.

Both cisplatin and oxaliplatin-treated mice displayed significant reductions in mechanical pain thresholds and also showed high levels of TRPA1 expression (11 fold and 6.7 fold, respectively). Pharmacological studies using TRPA1 antagonists have shown inhibition of inflammation and nerve injury-induced mechanical hyperalgesia in wild-type mice indicating that TRPA1 may contribute to mechanical transduction in some C-fiber nociceptors [61-64]. Interestingly, TRPA1 selective inhibitors failed to reverse inflammation-induced mechanical hyperalgesia in TRPA1-/- mice [65]. A potential compensatory mechanism might explain the masking contribution of this channel in TRPA-/- mice to maintain the development of mechanical hyperalgesia.

While its role in cold and mechanical sensation remains unclear, there is a growing body evidence that TRPA1 is a key chemical sensor and is directly activated by diverse chemicals and the products of cell and tissue injury [66]. Patients with avulsion injury and diabetic neuropathy having elevated TRPA1-immunoreactivity in DRG neurons [67] and with increased levels of reactive chemicals that are known to be TRPA1 activators [68,69], confirming that TRPA1 has a role in nociception. The behavioral phenotypes that we observe in platinum drug treated mice are consistent with elevated levels of expression of TRPV1 and TRPA1 mRNA in cisplatin-treated mice, and TRPA1 mRNA in oxaliplatin-treated mice, implicating TRPV1 and TRPA1 as key determinants in toxic neuropathy. Future genetic and pharmacological studies will determine the extent to which these channels contribute to thermal and chemical nociception in platinum drug-induced neuropathy.

### TRPV1 immunostaining following cisplatin and oxaliplatin-induced painful neuropathy

**Figure 3:**
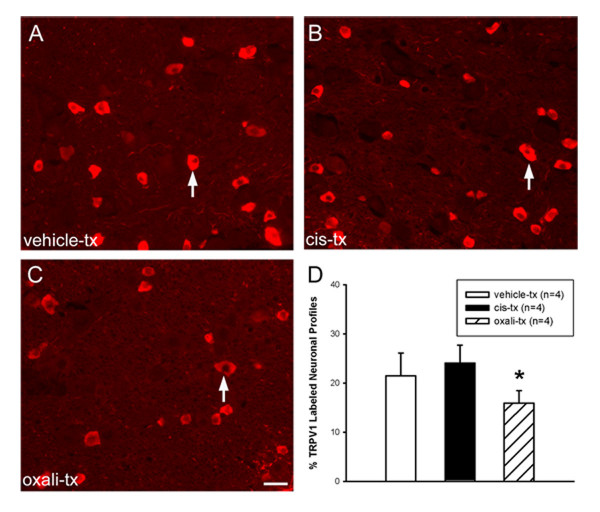
**TRPV1 immunoreactivity following platinum drug treatment**. Representative sections of TRPV1 immunoreactivity of TG neurons from **(A) **vehicle-, **(B) **cisplatin-, and **(C) **oxaliplatin- treated wild-type mice after 3 weeks treatment. Arrows indicate examples of labeled neuronal profiles. **(D) **Frequency histogram showing the percentage of labeled TRPV1 profiles in each group. Quantitative analysis of the proportion of TRPV1 immunopositive TG neuronal profiles stained as shown in **(A, B, and C) **from vehicle-, cisplatin-, and oxaliplatin-treated C57BL6J wild-type mice after 3 weeks treatment. Data represents mean ± SD, n = 4 mice/group, (*P < 0.05, two-way ANOVA, Scale bar = 25 μm).

To determine whether cisplatin and oxaliplatin induced changes in TRPV1 protein expression, immunostaining of TRPV1 protein was performed in the TG of wild-type mice after a 3-week platinum drug treatment protocol [48]. We assessed the average percentage of neuronal profiles expressing TRPV1 per section (Figure 3) and found an increase in the number of TRPV1 immunoreactive profiles in cisplatin-treated mice (24.06 ± 3.65%) compared to vehicle-treated controls (21.44 ± 4.65%), but this change was not statistically significant (P = 0.20) (Figure 3). There was a slight decrease in the number of TRPV1 immunoreactive neuronal profiles in oxaliplatin-treated mice (15.92 ± 2.52%; P = 0.04) (Figure 3). To characterize the population of TRPV1-protein positive neurons, we measured cell soma diameters in TRPV1-immunoreactive TG cells and observed average values of 16.66 ± 3.59 μm, 16.04 ± 3.23 μm, 16.99 ± 3.46 μm from vehicle-, cisplatin-, and oxaliplatin-treated population, respectively. These values also confirmed that TRPV1 is expressed in small neurons, consistent with their expression in nociceptors [70,71]. Thus, in this pain model, cisplatin treatment did not result in an overall change in the proportion of TRPV1 immunoreactive TG neurons.

Cancer patients undergoing chemotherapeutic therapy with platinum drugs can develop altered pain sensation resulting from sensory neuropathy [1]. In this study, we used models of cisplatin and oxaliplatin-induced neuropathy to investigate whether TRPV1, TRPM8, and TRPA1 mRNA expression were altered along with changes in thermal sensitivity observed in chemotherapeutic neuropathy. We show that cisplatin-treated mice developed heat hyperalgesia along with robustly increased levels of TRPV1 mRNA expression in TG, with no change in the proportion of TRPV1 immunopositive neurons. Furthermore, the increase in TRPV1 mRNA expression in TG in vivo mirrored that observed in DRG in vitro. We also confirm that cisplatin fails to influence thermal nociception in TRPV1-/- mice. Thus, our study provides evidence that up-regulation of TRPV1 at the transcriptional level is involved in mediating the heat hyperalgesia observed in mice with cisplatin-induced neuropathy.

While TRPV1 is known to play an important role in heat sensitivity, the mechanisms by which cisplatin-induced neuropathy affects the channel sensitization are unclear. Given the increased levels of TRPV1 mRNA that were found in the sensory nerve cell bodies, increased TRPV1 expression could result in enhanced TRPV1 protein trafficking to peripheral terminal nociceptors. Future cisplatin studies will evaluate the levels of TRPV1 protein expression in intraepidermal nerve fibers for evidence of cellular trafficking of TRPV1 to peripheral nociceptor terminals. Previous studies in inflammation and nerve injury have shown an increase of TRPV1 protein in DRG and unmyelinated axons in digital nerves [72,73]. Similarly, inflammation was also reported to increase the proportion of TRPV1 protein-positive neurons but not the mRNA level of the TRPV1 receptor in DRGs [74]. This transcription-independent mechanism of TRPV1 upregulation is found to be dependent on retrograde transport of NGF acting via p38 to increase TRPV1 translation in the cell body which results in increased transport of TRPV1 to peripheral terminals [74]. The participation of TRPV1 in hyperalgesia resulting from cisplatin-induced neuropathy might also involve channel phosphorylation by cellular kinases such as PKC, leading to enhanced TRPV1 sensitivity which contributes to pathological pain [66].

These findings reflect potentially varied mechanisms of TRPV1 channel sensitization but the nature and origin of these mechanistic differences remain to be established.

### Cisplatin treated-TRPV1 null and wild-type mice show similar reduced exploratory activity

**Figure 4:**
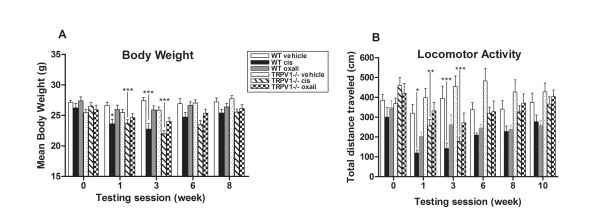
**Cisplatin-treated mice show reduced body weight and exploratory activity following each drug treatment cycle**. (A) Mean body weight of TRPV1^-/- ^and wild-type mice is reduced after cisplatin drug treatment. (B) Mean horizontal distance traveled in 20 min is reduced after each cisplatin treatment cycle from TRPV1^-/- ^and wild-type mice. Data represent the mean ± SEM, n = 7 mice/group (*P < 0.05, ***P < 0.01, ***P < 0.001, two-way ANOVA followed with Bonferroni post hoc analysis; two-tailed unpaired T test for genotypes).

Following platinum drug treatment animals were monitored for signs of toxicity with general observations. Ninety six mice were treated and all survived until the end of the study with the exception of one cisplatin-treated wild-type animal that died suddenly at the end of week four with no evidence of severe general toxicity. Neither genotype showed alterations of superficial and core body temperature or signs of nephrotoxicity (data not shown). Platinum-drug induced peripheral neurotoxicity is known to induce body weight loss. Mean body weight at baseline and post treatment was therefore evaluated for both genotypes. Both platinum drugs had similar temporal effects on body weight of both genotypes. Cisplatin-treated mice showed a slight but statistically significant reduction in mean body weight compared to vehicle-treated mice (Figure 4A). In contrast, oxaliplatin-treated mice only show a slight trend in lower body weight, but this reduction was not statistically significant (Figure 4A). Treated-mice from both genotypes gradually regained their initial body weight by 8 weeks after treatment. Animals with experimental induced neuropathies are frequently seen to have reduction in body weight that does not have a direct effect on their peripheral nerves [75,76]. Both platinum drugs had similar temporal effects on exploratory activity (Figure 4B) for mice of both genotypes. Cisplatin-treated mice showed slight decreases in body weight and locomotor activity, and treated-mice from both genotypes gradually returned to their basal level after drug cessation. Mice of both genotypes also maintained their muscle strength and had no evidence of drug effects on grip strength (data not shown). This mimics the situation in platinum-treated patients who develop a pure sensory neuropathy. The absence of cell death or axonal degeneration in sciatic nerve (data not shown) is also reflected in the reversibility of nociceptive behaviors seen in treated mice.

### Cisplatin-treated TRPV1-null mice exhibit reduced thermal hypersensitivity to noxious heat

**Figure 5:**
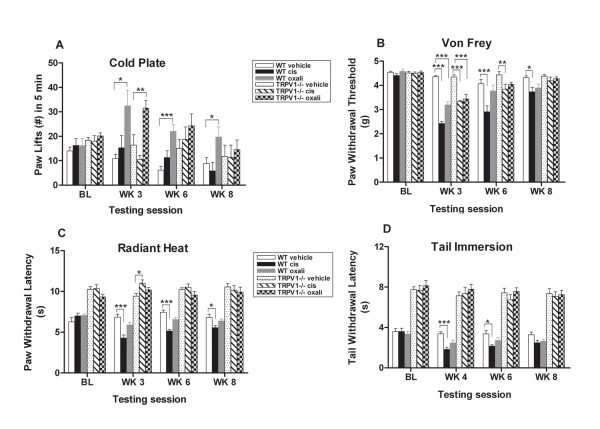
**Cisplatin-treated TRPV1-null mice exhibit alteredbehavioral responses to noxious heat stimuli**. **(A) **Oxaliplatin-treated TRPV1^-/- ^and wild-type mice show an increase in the number of paw lifts to noxious cold stimuli of -4.2°C in a cold plate assay. **(B) **Cisplatin and oxaliplatin-treated TRPV1^-/- ^and wild-type mice exhibit a decreased response threshold to punctate mechanical stimuli to the hind paw in von Frey assay. **(C) **Cisplatin-treated TRPV1^-/- ^mice show no noxious heat-evoked hyperalgesia in the hind paw in a radiant heat assay. In contrast, cisplatin-treated wild-type mice develop noxious heat-evoked hyperalgesia. **(D) **Cisplatin-treated TRPV1^-/- ^mice show no noxious heat-evoked hyperalgesia in a tail immersion test assay. In contrast, cisplatin-treated wild-type mice develop noxious heat-evoked hyperalgesia. Data represent the mean ± SEM, n = 7 mice/group (*P < 0.05; ***P < 0.001, two-way ANOVA followed with post hoc analysis; two-tailed unpaired T test for genotypes).

To determine whether the phenotypes that we observed at the molecular level are reflected in nocifensive behavior, we compared the behavioral responses of TRPV1^-/- ^and wild-type mice to different modalities of stimuli after a 3-week treatment protocol [48] with cisplatin, oxaliplatin or vehicle alone. Cold pain is one of the hallmark symptoms of oxaliplatin acute neuropathy. To determine whether ablation of TRPV1 alters the balance between hot and cold nociceptors, cold responses in TRPV1^-/- ^and wild-type mice were compared after 3 weeks of platinum drug treatment. Mice were placed on a cold plate calibrated at -4.2° ± 0.5°C and cold hypersensitivity was measured as the number of hind paw lifts over a 5-min duration. Consistent with the intact response of nociceptors to cold stimuli, we observed no inherent difference in the number of paw lifts between genotypes at baseline (Figure 5A). Oxaliplatin treatment induced a robust cold hypersensitivity in both genotypes. Oxaliplatin -treated TRPV1^-/- ^mice had a significant (2-fold) increase in the number of paw lifts compared to the vehicle-treated TRPV1^-/- ^controls (Figure 5A) and this change in cold response was also observed in the wild-type mice (Figure 5A). However, this cold hyperalgesia was only prolonged in the wild-type mice, remaining statistically significant out to 8 weeks (Figure 5A). Cold hypersensitivity did not persist in TRPV1^-/- ^mice. Cold induced hyperalgesia observed in oxaliplatin-treated mice therefore mimics the acute, painful dysesthesia reported by more than 80% of patients treated with oxaliplatin [77].

Previous studies have shown that TRPV1-null mice have normal basal mechanical sensitivity and normal mechanical hyperalgesic responses but impaired basal thermal sensitivity and thermal hyperalgesia following tissue inflammation [23,24]. Moreover, pharmacological evidence implicates TRPV1 involvement in mechanical hyperalgesia in models of inflammatory and neuropathic pain [78-80]. To determine whether TRPV1 contributes to the development of mechanical allodynia in this platinum drug-induced nerve injury model, mice of both genotypes were measured for mechanosensory response after platinum drug treatment. Mechanical response was evaluated by assessing the withdrawal thresholds to a calibrated von Frey filament that was applied to the hind paw. Consistent with the intact response of nociceptors to mechanical stimuli, there was no clear difference in the basal withdrawal thresholds to mechanical stimuli between genotypes (Figure 5B), as shown previously by others [23,81]. Platinum drug treatment consistently evoked marked mechanical allodynia for both genotypes. The withdrawal thresholds were significantly reduced for the cisplatin and oxaliplatin-treated TRPV1^-/- ^mice, 23% and 20%, respectively (Figure 5B). This reduction was greater for cisplatin and oxaliplatin-treated wild-type mice, 40% and 30%, respectively. While comparable hypersensitivity of mechanical responses was observed for both genotypes, slightly greater reduction in withdrawal thresholds was seen in cisplatin-treated wild-type mice at the later time point at week 8. This suggests that TRPV1 also participates in development of mechanical hyperalgesia in platinum drug-induced pain. Similar findings are also observed in TRPV1 null mice with intact mechanical hyperalgesia following inflammation, incision, and tumor-induced pain [23,82,83]. Interestingly, in acute pharmacological studies, TRPV1 antagonists appear to reduce mechanical hyperalgesia which could reflect a compensatory mechanism with TRPV1-/- mice masking the contribution of this channel [84].

To test whether TRPV1 is essential for thermal hyperalgesia in toxic neuropathy, TRPV1^-/- ^and wild-type mice were subjected to a Hargreaves radiant heat test after 3 weeks platinum drug treatment. A radiant heat source was applied to the hind paw and latency to paw licking or paw withdrawal was measured. TRPV1-null mice exhibited a significant 33% increase in mean baseline withdrawal latency compared to wild-type mice (Figure 5C). This difference remained relatively unchanged following treatment with platinum drugs. However, cisplatin-treated null mice showed transient hypalgesia after 3 weeks drug treatment. In contrast, cisplatin significantly reduced radiant heat-evoked withdrawal latency in wild-type mice by 40%, 30%, and 20% at weeks 3, 6, and 8, respectively (Figure 5C). Conversely, oxaliplatin induced no significant change in withdrawal latency for either genotype. Thus, TRPV1 plays a central role in the response to thermal pain and also significantly contributes to heat-evoked nociception after cisplatin treatment.

A tail immersion paradigm was also used to assess thermal hyperalgesia in TRPV1-null and wild-type mice after 3 weeks platinum drug treatment. The distal portion of the tail was immersed in a heated bath and the time of tail flick was recorded. TRPV1-null mice had a significant 50% increase of basal withdrawal latencies compared to wild-type mice (Figure 5D). Similar to the radiant heat test, cisplatin evoked little change in thermal response in TRPV1-null mice in the tail immersion test. In contrast, cisplatin treatment evoked consistent thermal hyperalgesia in the wild-type mice. Significant 45% and 35% decreases in withdrawal latency were detected for the cisplatin-treated wild-type mice compared to wild-type controls at weeks 4 and 6, respectively. Conversely, no such change was observed for oxaliplatin-treated mice in either genotype. A clear and robust impairment in heat evoked responses in both behavioral heat assays from cisplatin-treated TRPV1-null mice demonstrates that TRPV1 receptor is an essential mediator for thermal hyperalgesia in this cisplatin-induced painful neuropathy model.

## Conclusion

Despite their widespread clinical use, the molecular mechanisms through which platinum drugs mediate their effects on the nervous system remain unknown. Since there are distinct differences in the features of the pain associated with platinum compounds, our goal is to illustrate the utility of the recently established mouse model [48] in distinguishing between the two closely related platinum drugs in a way that is consistent with clinical observations. In the present study, we provide evidence that the up-regulations of TRPV1 and TRPA1 mRNA are coupled with enhanced heat and mechanical hypersensitivity in cisplatin-treated mice. In addition, the up-regulation of TRPA1 mRNA is observed with enhanced cold and mechanical hypersensitivity in oxaliplatin-treated mice. Thus, TRPV1 and TRPA1 are likely to have an important role in nociceptive processing in platinum drug-induced painful neuropathy. We also show that TRPV1^-/- ^mice display deficits in thermally evoked pain-related behaviors but are not insensitive to noxious heat. These results indicate that TRPV1 plays a crucial role in signaling heat-induced pain after cisplatin-induced nerve injury.

These results may further define the biology TRP channels and their role in platinum drug-induced neuropathy which could provide potential targets for treatment strategies to ameliorate neuropathic pain in these debilitating diseases.

## Methods

### Experimental animals and drugs treatment

This study was conducted with the approval of the Mayo Clinic Animal Care and Use Committee and in compliance with the regulations of the National Institutes of Health and the ethical guidelines of the International Association for the Study of Pain [85].

Male C57BL6J mice were used and were ordered at least two weeks prior to study to allow for housing adjustment. For TRPV1^-/- ^studies, B6.129X1-Trpv1^tm1Jul^/J mice were obtained from the Jackson Laboratory (Bar Harbor, ME) and propagated at Mayo Clinic Animal Husbandry. All behavioral studies for both genotypes were done on male mice. Mice were housed in cages of four in an enriched environment. Mice were 13-14 weeks old and weight was 24-26 g when the study began. All mice had free access to water and food and were exposed to a standard light cycle of 12 hours on and 12 hours off.

### Cisplatin and oxaliplatin-induced painful neuropathy model

After habituation to the test environment and baseline measurements of pain sensitivity, mice were randomized to three treatment groups of either cisplatin (2.3 mg/kg), oxaliplatin (3.0 mg/kg), or vehicle (0.9% saline). Mice were treated with daily intraperitoneal injection (i.p.) for 5 days, followed by 5 days of rest, for two cycles. Total cumulative doses of 23 mg/kg cisplatin and 30 mg/kg oxaliplatin over a total of ten injections were used as previously described [48].

### Behavioral assays

All the behavioral studies were performed with the operator were blinded to the genotype and status of drug treatment. The activity assay, cold plate assay, von Frey assay, radiant heat assay, and tail immersion assay were all conducted as previously described [48] and as described briefly below.

#### Activity monitoring

Monitoring of locomotor activity was carried out at baseline, during drug treatment at weeks 1, 2, 3, and after treatment at weeks 6, 8, and 10 using VersaMax Animal Activity Monitors (AccuScan Model RXYZCM-16, Columbus, OH). Although the VersaMax monitor collects information in 21 behavioral categories, we only analyzed distance traveled in 20 min, collected in 1-min intervals, collapsed into 10 2-min blocks, averaged and presented as group means ± SEM. Data were analyzed by a VersaMax Analyzer (AccuScan Model CDA-8, Columbus, OH).

#### Cold plate assay

For the assessment of cold hyperalgesia, we used a Peltier-cooled cold plate preset at -4.2° ± 0.2°C with a temperature sensor placed directly on the surface of the metal plate (TECA, Chicago, IL) and counted the number of paw lifts in 5 min. For each cold testing session, mice were brought to the testing room and allowed to acclimate for 10 min prior to being placed individually onto the cold metal surface enclosed in a clear plexiglass barrier of 8 cm W × 14 cm D × 14 cm H with a top cover. To insure the accuracy of paw lift counting, we videotaped each cold plate testing session using a video camcorder (Sony DCR-PC1000) and replayed in slow motion. The total number of brisk lifts of either hind paw, or jumping, was counted as a response to cold hyperalgesia. Movements associated with locomotion were distinct, involving coordinated movement of all four limbs and these were excluded. Mice were only tested once on any given test day to avoid any possible anesthetic or tissue damage effects that could be produced by repeated exposure to a cold surface. A maximum cut off time of 5 min was used to prevent tissue damage. Three separate trials were carried out on three separate days at base line and two separate trials during and after drug treatment at weeks 1, 3, 6, and 8 were averaged and presented as the mean number of paw lifts ± SEM.

#### Von Frey filament assay

For the measurement of mechanical allodynia, we used an Ugo Basile Dynamic Plantar Aesthesiometer (Stoelting, Wood Dale, IL) using the von Frey filament principle. Mice were placed under clear plastic boxes above a wire mesh floor that allowed full access to the paws. Acclimation and exploratory behavior were observed for up to two hours until mice became calm and close to motionless. Paw movement associated with locomotion or weight shifting was not counted as a withdrawal response. Paw withdrawal thresholds from eight trials, from both hind paws of each mouse, were averaged and recorded as mean ± SEM.

#### Radiant heat assay

For the assessment of paw thermal hyperalgesia, a radiant heat assay was conducted using a Plantar Ugo Basile apparatus (Stoelting, Wood Dale, IL). Mice can move freely in this apparatus on an elevated glass surface with plastic boxes above as the top cover. Mice are given a two hour acclimation period prior to testing until they become calm and motionless. Each hind paw was tested alternately with an interval of 5 min for four trials. Paw withdrawal latencies from eight trials, from both hind paws of each mouse, were averaged and recorded as mean ± SEM.

#### Tail immersion assay

For examining tail thermal hyperalgesia, a tail immersion test was conducted with the water bath preset at 50.5° ± 0.5°C. Latency to vigorous tail flick was recorded during three trials separated by at least 30 min and the three trials were averaged and presented as mean ± SEM. Cutoff time was set at 20 s, after which the mouse was removed regardless of behavioral response.

### Cell Culture for In Vitro Real-Time RT-PCR

Timed pregnant female Sprague-Dawley rats (Harlan, Madison, WI) were anesthetized with sodium pentobarbital and E15 rat pups were isolated for dissection of spinal ganglia of cervical, thoracic, lumbar, and sacral regions as previously described [50]. Only three experimental conditions were carried out at a time. For each experimental condition, 280-320 DRG from 7-8 rat pups were rapidly isolated, dissociated, and the neurons were plated onto rat tail collagen (BD Biosciences, Indianapolis, IN) in a coated 10 cm culture dish (Falcon™, Fisher Scientific) at a density of approximately 2 × 10^6 ^cells/dish. Each experimental condition was performed in triplicate. Cells were cultured for the first 24 h in Eagle's minimal essential medium (EMEM) medium along with other basic supplements. After 24 h, cultures were grown in the above medium supplemented with 1 × 10^-5 ^M 5-Fluorodeoxy-2-uridine and 1 × 10^-5 ^M uridine for 5 days to remove supporting cells. After 48 hours of equilibration with normal medium, cultures were randomized to one of three experimental conditions: 6.7 μM (2 μg/ml) cisplatin, 6.7 μM (2.6 μg/ml) oxaliplatin, or media alone. Cultures were harvested for RNA isolation after 6, 12, 24, and 48 h of drug treatment. Oxaliplatin (Sigma-Aldrich, St. Louis, MO) was dissolved in sterile distilled water (1 mg/ml). Pharmaceutical grade cisplatin (1 mg/ml) was obtained from Bristol-Myers Squibb Company (Princeton, NJ).

### Tissues Preparation for Standard and Real-Time RT-PCR

For standard RT-PCR, separate groups of four naïve adult male rats Sprague Dawley (Harlan, Madison, WI) and eight naïve adult male mice C57BL/6J (Jackson Lab, Maine) were deeply anesthetized with sodium pentobarbital (50 mg/kg i.p.) and left and right TG and DRG tissues were quickly dissected from each animal. For real-time RT-PCR, separate groups of twenty seven wild-type mice, nine from each treatment group (cisplatin, oxaliplatin, vehicle), were anesthetized with sodium pentobarbital (50 mg/kg i.p.) 24 h after completion of a 3-week drug treatment protocol, then left and right TG tissues were rapidly dissected and removed from each animal. The nine mice from each treatment group were divided three groups of three mice each and TG from these groups were pooled and processed for triplicate samples. The collected TG tissues were quickly placed on dry ice and stored at -80°C prior to RNA isolation. It was necessary to pool the tissue to prevent significant loss of RNA during the extraction procedure.

### In Vitro and In Vivo RNA Extraction

For in vitro real-time RT-PCR, total RNA was isolated from rat DRG using a column homogenizer (Qiagen Shredder, Qiagen Inc., CA) and an RNeasy Mini Plus Kit (Qiagen Inc., CA) according to the manufacturer's directions. For standard RT-PCR and in vivo real-time RT-PCR, total RNA was isolated from rat and mouse DRG and TG tissues using a tissue homogenizer (Tenbroeck, Fisher Scientific) and an RNeasy Lipid Tissue Mini Kit (Qiagen Inc., CA) according to the manufacturer's directions. Samples were DNase treated with RNA-free DNAse (Qiagen Inc., CA) and quantified on a NanoDrop 1000 spectrophotometer (NanoDrop Technologies, Wilmington, DE, USA) prior to proceeding with standard and real-time RT-PCR. RNA quality and integrity, which can substantially affect the outcome of real-time RT-PCR measurements, was verified by electrophoretic analysis using a Bioanalyzer 2100 (Agilent Technologies, Palo Alto, CA) by Mayo Molecular Core Facility and all samples had RNA integrity (RIN) values of 8.5-9.5. RIN values of 8 or higher are considered perfect for downstream RNA applications [86].

### Standard RT-PCR

To detect TRPV1, TRPM8, and TRPA1 mRNA expression in sensory neurons, TG and DRG tissues from control rats and mice were isolated for RNA extraction followed by RT-PCR and gel electrophoresis. Total RNA was reverse transcribed into cDNA using Moloney Murine Leukemia Virus Reverse Transcriptase with oligo (dT) and random hexamers as primer (iScript cDNA Synthesis Kit, BioRad, CA). The reverse transcription (RT) reaction was performed in a 40 μl solution of 8 μl 5× iScript Reaction Mix, 2 μl iScript Reverse Transcriptase, and 0.2 μg RNA. Negative controls (without reverse transcriptase) were included. The RT condition was performed at 25°C for 5 minutes, 42°C for 30 minutes, and 85°C for 5 minutes using a MyIQ™ thermal cycler (BioRad, CA). The synthesized cDNA products were then used directly as templates for PCR amplification. The PCR reaction was performed in a 25 μl solution containing 20 μl of PCR Supermix (Platinum Supermix High Fidelity, Invitrogen), with 5 picomoles primers, and cDNA (20 ng - 200 ng). PCR amplification was performed for 30 cycles. Following an initial hot start of 2 minutes at 94°C, each cycle consisted of 1 min of denaturation at 94°C, 1 min of annealing at 56°C, and 1 min of extension at 72°C using a MyIQ™ thermal cycler (BioRad, CA). Each sample was run in duplicate. The amplified PCR products were separated by electrophoresis on a 2% agarose gel containing ethidium bromide and were visualized with UV illumination.

### Taqman Real-Time RT-PCR

Reverse transcription of 400 ng mRNA was performed with the iScript cDNA Synthesis Kit (BioRad, CA) according to manufacturer's instructions as described above. Taqman PCR assays were performed in triplicate on samples of cDNA from rat DRG neurons (in vitro real-time PCR) or from mouse TG tissues (in vivo real-time PCR) and were carried out in 96-well optical plates (BioRad, CA) using a MyIQ™ thermal cycler (BioRad, CA). Each real-time RT-PCR was repeated with at least an additional independent run with different cells or tissue cDNA samples. Real-time PCR reactions consisted of cDNA product (20 ng - 200 ng), 10 μM primer, 10 μM Taqman probe, and 12.5 μl 2× BioRad IQ SuperMix (#170-8860, IQ Supermix, BioRad, CA) in a total volume of 20 μl. Amplification was performed for 51 cycles. Following an initial hot start of 3 minutes at 95°C, each cycle consisted of 15 s of denaturation at 95°C, and 1 min of annealing at 60°C. Control reactions included PCR reactions on DNase RNA (without RT) and reactions run without templates to test for contamination. Oligonucleotide probes were serially diluted in nuclease-free water to produce a standard curve relating threshold cycle to template copy number. The threshold cycle, which represents the PCR cycle at which an increase in reporter fluorescence above background is first detected, was determined by the software (MyIQ, BioRad, CA) based on the generated standard curves. All essays showed a linear correlation between cycle threshold and amplicon in serial dilution experiments. Only PCR reactions run with efficiency in the range of 98% to 100% were included for calculations. The relative quantification of real time RT-PCR products was performed using the 2^-ΔΔCT ^method [87]. The fold change of mRNA expression levels of target genes TRPV1, TRPM8, and TRPA1 were normalized to 28S RNA as the reference gene, followed by normalization to controls. Data is presented as fold induction of platinum-drug treated cells or tissues compared to the expression level in vehicle-treated cells or tissues. Each target gene was considered to be differentially expressed when the Livak's calculation yielded an average fold difference of at least ± 2.0 compared to reference gene.

### Oligonucleotides

For standard RT-PCR, the primers specific for rat and mouse TRPV1, TRPM8, TRPA1, β-actin, and 28S RNA were designed using Primer Express software (Applied Biosystems, Foster City, USA) (Table 1). For real-time RT-PCR, the primers and Taqman probes specific for rat and mouse TRPV1, TRPM8, TRPA1, and 28S RNA were designed using the Roche Universal Library Assay Design Center (Roche Applied Science, Indianapolis) (Table 2). Each primer set produced nucleotide amplicon corresponding to the specific Roche probe catalog from universal library probes (Roche Applied Science, Indianapolis) (Table 2). Primers used in this study are listed in table 1 and table 2 with gene name, accession number, primer orientation, sequence (5' to 3'), and product size in base pairs.

**Table 1:** Oligonucleotide Sequences of Primers Used for Standard RT-PCR

Rat TRPV1	NM_031982	Forward	GGTGTGCCTGCACCTAGC		107 bp
		Reverse	CTCTTGGGGTGGGGACTC		

Rat TRPM8	NM_134371.2	Forward	GCCCAGTGATGTGGACAGTA		64 bp

		Reverse	GGACTCATTTCCCGAGAAGG		

Rat TRPA1	NM_207608.1	Forward	ATTTGCGGCCTGAGTTTTT		68 bp

		Reverse	TCCATCATTGTCCTCATCCA		

Rat 28S RNA	V01270	Forward	GACAGGTTAGTTTTACCCTACTGATGA		74 bp

		Reverse	CCTGCGGTTCCTCTCGTA		

Rat β-actin	NM_031144.2	Forward	CCCGCGAGTACAACCTTCT		72 bp

		Reverse	CGTCATCCATGGCGAACT		

Mouse TRPV1	NM_001001445	Forward	TTGGATTTTCCACAGCCGTAGT		155 bp

		Reverse	GAACTTGAACAGCTCCAGACATGT		

Mouse TRPM8	NM_134252	Forward	CCAAATCACAATGAGGTCACAGC		185 bp

		Reverse	CTTCAGGTGTAAAGTCCTCTGTACACTT		

Mouse TRPA1	NM_177781	Forward	ATTGTTCTCATGAACTTACTGATTGGCT		186 bp

		Reverse	CCTGGGTCTATTTGGATACACGAT		

Mouse β-actin	NM_007393	Forward	ACCCACACTGTGCCCATCTA		342 bp

		Reverse	GCCACAGGATTCCATACCCA		

**Table 2:** Oligonucleotide Sequences of Primers Used for Real-Time RT-PCR

Rat TRPV1	NM_031982	Forward	GGTGTGCCTGCACCTAGC		107 bp
		Reverse	CTCTTGGGGTGGGGACTC		

Rat TRPM8	NM_134371.2	Forward	GCCCAGTGATGTGGACAGTA		64 bp

		Reverse	GGACTCATTTCCCGAGAAGG		

Rat TRPA1	NM_207608.1	Forward	ATTTGCGGCCTGAGTTTTT		68 bp

		Reverse	TCCATCATTGTCCTCATCCA		

Rat 28S RNA	V01270	Forward	GACAGGTTAGTTTTACCCTACTGATGA		74 bp

		Reverse	CCTGCGGTTCCTCTCGTA		

Mouse TRPV1	AB180097.1	Forward	ACCACGGCTGCTTACTATCG		68 bp

		Reverse	TCCCCAACGGTGTTATTCAG		

Mouse TRPM8	NM_134252	Forward	TCAGATACACAGAGATCCTTCTGC		75 bp

		Reverse	GGCTCCCTCGAAGGACAT		

Mouse TRPA1	NM_17778.21	Forward	CCATGACCTGGCAGAATACC		73 bp

		Reverse	TGGAGAGCGTCCTTCAGAAT		

Mouse 28S RNA	X00525.1	Forward	GGCCACTTTTGGTAAGCAGA		143 bp

		Reverse	GCGGATTCCGACTTCCAT		

### Immunohistochemical staining of TRPV1 in Trigeminal Ganglia

Male mice were transcardially perfused (n = 4 for each group) with 4% paraformaldehyde with 01% pircric acid in 0.1 M phosphate buffer (PB) at 4°C and the trigeminal ganglia (TG) on both sides were removed and placed overnight in 30% sucrose-PB. Using a cryostat (Microm, Germany), the TG were sectioned at 8 μm through the short axis and mounted on 3 well Teflon-coated, gelatin dipped slides (Electron Microscopy Sciences, Hatfield, PA). For accuracy each ganglia was measured before cutting and sections were collected at 6 equally spaced intervals throughout the entire length. The sections were immunostained for TRPV1 by first permeabilizing the membrane by rinsing in 0.1 M phosphate buffered saline (PBS) with 0.2% triton X-100 for 10 min followed by blocking for 1 hr at room temperature (RT) in 10% normal goat serum in PBS. Sections were incubated overnight at RT in goat anti-TRPV1 (1:1000, catalog no. sc-12498, Santa Cruz Biotechnology, Santa Cruz, CA). Following 3 rinses in PBS, the sections were placed for 1 h in donkey anti-goat IgG cyanine 3 (Cy3) conjugate (1:400, Jackson ImmunoResearch, West Grove, PA). After rinsing again in PBS the sections were coverslipped with Vectashield (Vector Labs, Burlingame, CA). The specificity of this antibody was previously assessed by incubating sections in antiserum pre-absorbed with the corresponding peptide (100 μg peptide/1 ml antiserum). None of the sections immunostained with this solution showed positive immunofluorescence [88]The total number of neuronal profiles in each section was determined by counterstaining adjacent sections with 1% cresyl violet.

### Analysis of TRPV1 staining in the Trigeminal Ganglia

The study was blinded so the investigator was unaware of what treatment group was being analyzed. After staining, color digitized images of sections to be analyzed (24 bits per pixel), were captured using the 20× objective on an Olympus BX51 microscope with Cy3 filter cube and an attached Spot RT digital camera (Diagnostic Instruments, Sterling Heights, MI). Camera exposures were adjusted automatically by Spot software (version 3.1) and brightness and gamma corrections were made manually to achieve the best signal-to-noise ratios. Percentages of TRPV1-labeled neuronal profiles were calculated by dividing the number of labeled profiles by the total number of TG profiles in the section *x *100. An average was then obtained for the 6 sections of tissue analyzed for each animal.

The diameters of TG neurons were calculated by summing the length and width of those neurons with a visible nucleus and dividing by two. Approximately 200 neurons were measured from the 4 mice in each group. Data are presented as an average ± SD.

### Statistical analyses

Immunohistochemical data are presented as the mean ± standard deviation (SD), all other data are expressed as mean ± standard error of the mean (SEM). Results were illustrated and analyzed using Graphpad Prism version 4 (Graphpad Software, San Diego, USA). Statistical analyses on behavioral studies were performed using two-way ANOVA with drug treatments and time as independent variables to examine the differences in response across treatment groups. Follow-up analysis was conducted using the Bonferroni test. For comparisons between wild type and TRPV1 null mice, two-tailed unpaired Student's *t *tests were used. For immunohistochemical data, differences among groups were analyzed with a two-way ANOVA. *P *value of less than 0.05 is considered statistically significant.

## Competing interests

The authors declare that they have no competing interests.

## Authors' contributions

LET conceived and designed the study, conducted the molecular and behavioral studies, performed the data analysis, interpreted the results, and wrote the manuscript. AJB generated the TRPV1-/- mouse colony, participated in the interpretation of the results and reviewing of the final manuscript. SCM conducted the immunohistochemical studies, participated in the interpretation of the results and reviewing of the final manuscript. CLL participated in the interpretation of the results. PAL participated in the design of the study. AJW participated in the study design, interpretation of the results and reviewing of the final manuscript. All authors have read and approved the final manuscript.
